# Importance of Discriminative Measurement for Radon Isotopes and Its Utilization in the Environment and Lessons Learned from Using the RADUET Monitor

**DOI:** 10.3390/ijerph17114141

**Published:** 2020-06-10

**Authors:** Chutima Kranrod, Yuki Tamakuma, Masahiro Hosoda, Shinji Tokonami

**Affiliations:** 1 Institute of Radiation Emergency Medicine, Hirosaki University, Hirosaki, Aomori 036-8564, Japan; kranrodc@hirosaki-u.ac.jp (C.K.); tamakuma@hirosaki-u.ac.jp (Y.T.); m_hosoda@hirosaki-u.ac.jp (M.H.); 2 Natural Radiation Survey and Analysis Research Unit, Department of Nuclear Engineering, Faculty of Engineering, Chulalongkorn University, Bangkok 10330, Thailand; 3 Graduate School of Health Sciences, Hirosaki University, Hirosaki 036-8564, Aomori, Japan

**Keywords:** radon–thoron discrimination, RADUET, radon, thoron

## Abstract

Radon (^222^Rn) and thoron (^220^Rn), sources of natural background radiation, have been the subjects of long-standing studies, including research into radon and thoron as major causes of lung cancer at domestic and international levels. In this regard, radon and thoron measurement studies have been widely conducted all over the world. Generally, the techniques used relate to passive nuclear track detectors. Some surveys have shown that passive monitors for radon are sensitive to thoron, and hence some measured results have probably overestimated radon concentrations. This study investigated radon and thoron measurements in domestic and international surveys using the passive radon–thoron discriminative monitor, commercially named RADUET. This paper attempts to provide an understanding of discriminative measurements of radon isotopes and to present an evidence-based roadmap.

## 1. Introduction

Radon (^222^Rn) is a noble gas generated from materials including radium (^226^Ra) (a decay product of uranium (^238^U)) such as rocks, soil, water and building materials. Radon is well known as the second leading cause of lung cancer after tobacco smoking [1]. According to an epidemiological investigation by Darby et al. [2], consecutive exposure to 100 Bq m^−3^ of radon results in a 16% excess relative risk of lung cancer. The World Health Organization recommended that a radon concentration of 100 Bq m^−3^ should be used as a national reference value and stated that this value was justified from a public health effect perspective [1]. Even if it cannot be implemented, the chosen reference level should not exceed 300 Bq m^−3^ which corresponds to 10 mSv of effective doses per year based on the calculations by the International Commission on Radiological Protection (ICRP) [3]. The ICRP also encouraged use of the reference level of 100−300 Bq m^−3^ [4]. Besides this, a new dose conversion factor for radon was proposed by the ICRP [5] according to the latest epidemiological studies and a dosimetric model, and this factor is approximately two times higher than the commonly used value of the United Nations Scientific Committee on the Effects of Atomic Radiation (UNSCEAR) [6]. Thus, it is increasingly important to accurately measure radon and to evaluate the dose attributed to radon and its progenies from the viewpoint of radiation protection for humans.

Many radon surveys have been conducted in countries all over the world to investigate radon levels [7,8,9,10,11,12,13]. Generally, passive radon monitors are used for large-scale surveys because their operation is quiet and low-cost. However, it has been reported that some have a high sensitivity to thoron (^220^Rn), which is a radioisotope of radon, and the measurement results might have been affected by the existence of thoron [14,15,16]. Passive radon monitors which had been shown to have a high sensitivity to thoron were used for a nationwide radon survey in some countries [8,9,10,17]. It is necessary to pay attention to those reports in which the possible presence of thoron would significantly bias results for radon measurements. From the viewpoint of radiation protection, thoron has been historically ignored because of its half-life (55.6 s) and difficulties in measurement and calibration. It should be noted, however, that the quantity of thoron can be larger than that of radon in some areas, for example in Yangjang, China [18]. If a passive radon monitor cannot discriminatively measure radon and thoron, measurement results will be overestimated, and they cannot be corrected due to no significant relationship between radon and thoron concentrations [15,19]. Therefore, radon and thoron should be discriminatively measured for an accurate dose assessment and for future lung cancer risk assessment due to indoor radon [6].

This article presents a summary of the passive radon–thoron discriminative monitors known as RADUET and their applications in studies worldwide.

## 2. Summary of RADUET

**Figure 1:**
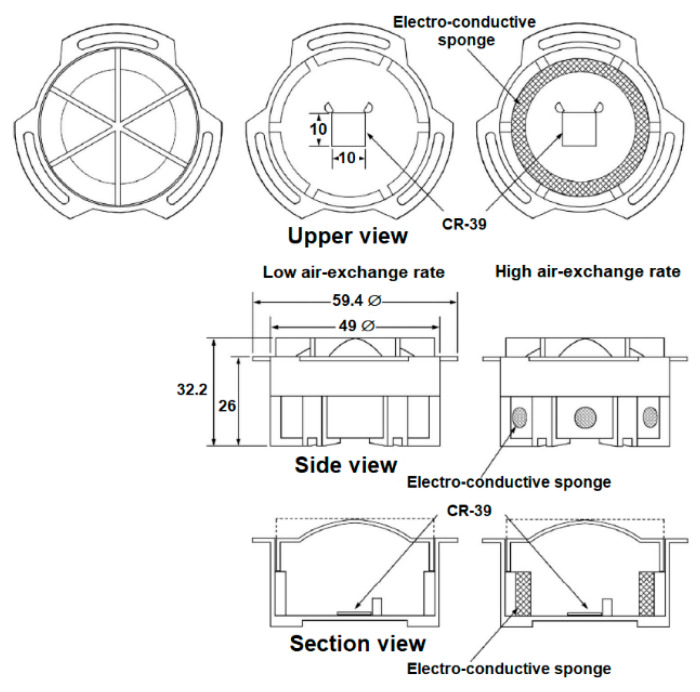
Schematic views of the RADUET monitor (unit: mm).

The RADUET is a passive integrated radon–thoron discriminative monitor, which was developed by Tokonami et al. [20]. Schematic drawings of the RADUET monitor are shown in Figure 1. It has double plastic chambers with different air-exchange rates or air-diffusion rates. A CR-39 (the allyl diglycol carbonate) detector, which is used to detect alpha particles emitted from radon, thoron and their progenies, is placed at the bottom of each chamber with sticky clay. The low air-exchange rate chamber is made of electro-conductive plastic with an inner volume of ~3.0 × 10^−5^ m^3^. The high air-exchange rate chamber is also made of the same plastic material, but it has six holes in the wall and has an electroconductive sponge covering the holes to prevent radon and thoron decay products and aerosols from going into the chamber. Thus, radon gas can diffuse into the low air-exchange rate chamber through an invisible gap between the lid and the bottom of the chamber. Subsequently, this gap functions as a high air-diffusion rate barrier; due to its very short half-life (55.6 s), only a very small amount of thoron goes into the chamber compared to the amount for radon with a longer half-life (3.82 d). Additionally, both radon and thoron can get into the high air-exchange rate chamber. The air-exchange rates of the low and high air-diffusion rate chambers are 0.7 h^−1^ and 10 h^−1^, respectively [21]. The air-exchange rates of RADUET chambers differ by about two orders of magnitude. Correspondingly, the difference of track density between the two CR-39s detectors from each chamber makes it possible to discriminate between radon and thoron.

For analysis of the CR-39 detectors from the RADUET, they are chemically etched in concentrated base solution according to the manufacturers’ protocols. For example, CR-39 detectors manufactured by Radosys Ltd. (Budapest, Hungary) have to be etched with a 6.25 M NaOH solution at 90 °C for 6 h, while CR-39 detectors manufactured by Nagase Landauer, Ltd. (Ibaraki, Japan) have to be etched with a 6 M NaOH solution at 60 °C for 24 h. After that, the tracks formed on CR-39 are counted with an automatic reading system or optical microscope and image software. For calculating radon and thoron concentrations, the obtained total track densities are replaced into the following equations [22]:

(1) $${\bar{C}}_{\mathrm{Rn}}=\left(d_{L}-\bar{b}\right)\times \;\frac{f_{\mathrm{Tn}2}}{t\times \left(f_{\mathrm{Rn}1}\times f_{\mathrm{Tn}2}-f_{\mathrm{Rn}2}\times f_{\mathrm{Tn}1}\right)}-\left(d_{H}-\bar{b}\right)\times \frac{f_{\mathrm{Tn}1}}{t\times \left(f_{\mathrm{Rn}1}\times f_{\mathrm{Tn}2}-f_{\mathrm{Rn}2}\times f_{\mathrm{Tn}1}\right)}$$

(2) $${\bar{C}}_{\mathrm{Tn}}=\left(d_{H}-\bar{b}\right)\times \frac{f_{\mathrm{Rn}1}}{t\times \left(f_{\mathrm{Rn}1}\times f_{\mathrm{Tn}2}-f_{\mathrm{Rn}2}\times f_{\mathrm{Tn}1}\right)}-\left(d_{L}-\bar{b}\right)\times \frac{f_{\mathrm{Rn}2}}{t\times \left(f_{\mathrm{Rn}1}\times f_{\mathrm{Tn}2}-f_{\mathrm{Rn}2}\times f_{\mathrm{Tn}1}\right)}$$

where ${\bar{C}}_{\mathrm{Rn}}$ and ${\bar{C}}_{\mathrm{Tn}}$ are the mean concentrations of radon and thoron during the exposure period in Bq m^−^^3^. *d*_L_ and *d*_H_ are the total alpha track densities (track m^−2^) taken from the CR-39 detectors of low and high air-exchange rate chambers. *f*_Rn1_ and *f*_Tn1_ are the radon and thoron calibration coefficients for the low air-exchange rate chamber in tracks m^−^^2^ kBq^−^^1^ m^3^ h^−^^1^. *f*_Rn2_ and *f*_Tn2_ are the radon and thoron calibration coefficients for the high air-exchange rate chamber in tracks m^−^^2^ kBq^−^^1^ m^3^ h^−^^1^. *t* is the exposure time in hours and *b* is the background track density of the CR-39 detector in tracks m^−^^2^.

It should be noted that the low air-exchange rate chamber limits diffusion of thoron into the chamber, therefore, *f*_Rn1_ >> *f*_Tn1_. The high air-exchange rate chamber is designed such that both radon and thoron can diffuse into the chamber easily, and *f*_Rn2_ ~ *f*_Tn2_.

To determine the calibration coefficients, RADUETs should be calibrated at a reference laboratory. The calibration coefficients are estimated by a correlation between the track density and the time-integrated radon and thoron concentrations from three or four exposure levels of radon and thoron [23]. In the calibration procedure of the radioactive gas monitor, most RADUET monitors are calibrated using a secondary method based on a calibration via a reference monitor. Thus, five to ten monitors, depending on the size of the calibration chamber, are placed in the middle of the chamber for each reference exposure condition. The introduction level of radon and thoron in each calibration chamber should be assigned to three or four different levels for the time-integrated radon and thoron concentrations such as ~500 kBq h m^−3^, ~1000 kBq h m^−3^, ~2000 kBq h m^−3^ and ~3000 kBq h m^−3^. These calibrations are intended to obtain traceability to primary standards of radon isotopes and to test linearity of monitor response over the whole range of interest of exposure values. Throughout the calibration experiments, concentration of radon isotopes in the chamber is continuously monitored with active measurement equipment which has itself been compared with a standard for radon isotopes. Moreover, environmental parameters, such as humidity, pressure and temperature in the calibration chamber should be continuously measured and constantly observed. After exposure, the CR-39 detectors from RADUET are chemically etched and afterwards the number of tracks on the CR-39 is counted as used for measurement samples. In addition, the background noise from five or ten CR-39 detectors that have not been exposed to radon and thoron and have been processed under the same chemical etching and counting conditions are measured at the same time as the calibration. Subsequently, the four calibration curves are plotted as the time-integrated radon or thoron exposure (on the X axis) versus the track density (on the Y axis) for the high and low air-exchange rates of RADUET. Thus, *f*_Rn1,_ *f*_Tn1,_ *f*_Rn2_ and *f*_Tn2_ are given by fitting each calibration curve using linear regression [20]. The slope of the linear line of each calibration curve is the calibration coefficient or calibration factor between the density of the tracks per unit of time (tracks m^−2^ h^−1^) and the activity concentration (kBq m^3^) of the reference atmosphere.

Moreover, based on the fact of the RADUET that concentration in one chamber depends on the other, the calculation procedure for the decision threshold and detection limits are estimated in the following manner [22]. The decision thresholds of the average radon concentration $\left({\bar{C}}_{\mathrm{Rn}}^{*}\right)$ and the average thoron concentration $\left({\bar{C}}_{\mathrm{Tn}}^{*}\right)$ are obtained from Equations (1) and (2) and their standard uncertainty for ${\tilde{C}}_{\mathrm{Rn}}=0$, $\tilde{u}\left(d_{L}\right)=0$, ${\tilde{C}}_{\mathrm{Tn}}=0$ and $\tilde{u}\left(d_{H}\right)=0$. These yield Equations (3) and (4). (It should be noted that α = 0.05 and k_1−α_ = 1.65 are often selected by default.)

(3) $${\bar{C}}_{\mathrm{Rn}}^{*}=\;k_{1-\alpha }\cdot \tilde{u}\left(0\right)=k_{1-\alpha }\sqrt{\begin{array}{c} \omega_{1}^{2}u^{2}\left(\bar{b}\right)-2\omega_{1}\omega_{2}u^{2}\left(\bar{b}\right)+\omega_{2}^{2}(u^{2}\left(d_{H}\right)+u^{2}\left(\bar{b}\right))+\frac{\left(d_{H}^{2}-2\bar{b}d_{H}+{\bar{b}}^{2}\right)\omega_{2}^{2}}{\omega_{1}^{2}}u^{2}\left(\omega_{1}\right)\\ +{\left(-d_{H}+\bar{b}\right)}^{2\;}u^{2}\left(\omega_{2}\right) \end{array}}$$

(4) $${\bar{C}}_{\mathrm{Tn}}^{*}=k_{1-\alpha }\cdot \tilde{u}\left(0\right)=k_{1-\alpha }\sqrt{\begin{array}{c} \omega_{3}^{2}u^{2}\left(\bar{b}\right)-2\omega_{3}\omega_{4}u^{2}\left(\bar{b}\right)+\omega_{4}^{2}(u^{2}\left(d_{L}\right)+u^{2}\left(\bar{b}\right))+\frac{\left(d_{L}^{2}-2\bar{b}d_{L}+{\bar{b}}^{2}\right)\omega_{4}^{2}}{\omega_{3}^{2}}u^{2}\left(\omega_{3}\right)\\ +{\left(-d_{L}+\bar{b}\right)}^{2\;}u^{2}\left(\omega_{4}\right) \end{array}}$$

The detection limit of the average radon $({\bar{C}}_{\mathrm{Rn}}^{\#}$) and thoron concentration $\left({\bar{C}}_{\mathrm{Tn}}^{\#}\right)$ are calculated as given in Equations (5) and (6). (It should be noted that α = β = 0.05 and k_1−α_ = k_1−β_ = 1.65 are often selected by default.)

(5) $${\bar{C}}_{\mathrm{Rn}}^{\#}={\bar{C}}_{\mathrm{Rn}}^{*}+k_{1-\beta }\cdot \tilde{u}\left[{\bar{C}}_{\mathrm{Rn}}^{\#}\right]={\bar{\;C}}_{\mathrm{Rn}}^{*}+k_{1-\beta }\sqrt{\begin{array}{c} \omega_{1}^{2}[u^{2}\left(d_{L}\right)+u^{2}\left(\bar{b}\right)]-2\omega_{1}\omega_{2}u^{2}\left(\bar{b}\right)+\omega_{2}^{2}[(u^{2}\left(d_{H}\right)+u^{2}\left(\bar{b}\right)]\\ +\frac{\left(d_{H}^{2}-2\bar{b}d_{H}+{\bar{b}}^{2}\right)\omega_{2}^{2}+{\tilde{C}}_{\mathrm{Rn}}\left(2d_{H}-2\bar{b}\right)\omega_{2\;}+{\tilde{C}}_{\mathrm{Rn}}^{2}}{\omega_{1}^{2}}u^{2}\left(\omega_{1}\right)+{\left(-d_{H}+\bar{b}\right)}^{2\;}u^{2}\left(\omega_{2}\right) \end{array}}$$

(6) $${\bar{C}}_{\mathrm{Tn}}^{\#}={\bar{C}}_{\mathrm{Tn}}^{*}+k_{1-\beta }\cdot \tilde{u}\left[{\bar{C}}_{\mathrm{Tn}}^{\#}\right]={\bar{C}}_{\mathrm{Tn}}^{*}+k_{1-\beta }\sqrt{\begin{array}{c} \omega_{3}^{2}[u^{2}\left(d_{H}\right)+u^{2}\left(\bar{b}\right)]-2\omega_{3}\omega_{4}u^{2}\left(\bar{b}\right)+\omega_{4}^{2}[(u^{2}\left(d_{L}\right)+u^{2}\left(\bar{b}\right)]\\ +\frac{\left(d_{L}^{2}-2\bar{b}d_{L}+{\bar{b}}^{2}\right)\omega_{4}^{2}+{\tilde{C}}_{\mathrm{Tn}}\left(2d_{L}-2\bar{b}\right)\omega_{4\;}+{\tilde{C}}_{\mathrm{Tn}}^{2}}{\omega_{1}^{2}}u^{2}\left(\omega_{3}\right)+{\left(-d_{L}+\bar{b}\right)}^{2\;}u^{2}\left(\omega_{4}\right) \end{array}}$$

Here $\omega_{1}=\frac{f_{\mathrm{Tn}2}}{t(f_{\mathrm{Rn}1}\cdot f_{\mathrm{Tn}2}-f_{\mathrm{Rn}2}\cdot f_{\mathrm{Tn}1})},\omega_{2}=\frac{f_{\mathrm{Tn}1}}{t(f_{\mathrm{Rn}1}\cdot f_{\mathrm{Tn}2}-f_{\mathrm{Rn}2}\cdot f_{\mathrm{Tn}1})},\omega_{3}=\frac{f_{Rn1}}{t(f_{\mathrm{Rn}1}\cdot f_{\mathrm{Tn}2}-f_{\mathrm{Rn}2}\cdot f_{\mathrm{Tn}1})},$ and $\;\omega_{4}=\frac{f_{\mathrm{Rn}2}}{t(f_{\mathrm{Rn}1}\cdot f_{\mathrm{Tn}2}-f_{\mathrm{Rn}2}\cdot f_{\mathrm{Tn}1})}$. ${\tilde{C}}_{\mathrm{Rn}}\;\mathrm{and}\;{\tilde{C}}_{\mathrm{Tn}}$ are the true values of the average radon and thoron concentrations. $\tilde{u}\left(d_{L}\right)\;\mathrm{and}\;\tilde{u}\left(d_{H}\right)$ are the standard uncertainties of the estimators of ${\tilde{C}}_{\mathrm{Rn}}\;\mathrm{and}\;{\tilde{C}}_{\mathrm{Tn}}$. $\tilde{u}\left[{\bar{C}}_{\mathrm{Rn}}^{\#}\right]\;\mathrm{and}\;\tilde{u}\left[{\bar{C}}_{\mathrm{Tn}}^{\#}\right]$ are the standard uncertainties of detection limits of average radon and thoron concentrations associated with measurement results. 

Based on the calculation procedure given above, the detection limits for the typical measurement conditions have been estimated in the literature to be 3 Bq m^−3^ for radon and 14 Bq m^−3^ for thoron [20]. Moreover, the detection limits for the high background area condition have been assessed to be 10 Bq m^−3^ for radon and 20 Bq m^−3^ for thoron [24].

## 3. Lessons Learned on Radon and Thoron Measurement by the RADUET

Radon and thoron simultaneous measurements using a passive detector have been carried out for indoor environments in many countries [15,25,26,27,28,29]. The RADUET has been one of the most favored radon and thoron discriminative detectors used for both small and large national surveys. Furthermore, there are many studies using the RADUET for such applications as validation of the scanner-based radon track detector evaluation system [30], radon and thoron exhalation rates from building materials [31] and performance testing of the effects of environmental parameters on passive detectors [32].

This section points out the value of discriminating thoron from radon in measuring radon isotopes by describing regional and national surveys which have been made in various countries.

In China, RADUETs were used to determine the indoor concentrations of radon and thoron at high natural background radiation areas in Yangxi and Yangdong Districts [18,33]. RADUETs were placed at the distance of ~0.3 m from the wall and ~1.8 m from the floor. Indoor radon and thoron concentrations were measured for six months at 59 dwellings and wide variations were seen from house to house. The mean indoor radon concentration was 124 ± 78 Bq m^−3^ (range from 27–476 Bq m^−3^) and the mean indoor thoron concentration was 1247 ± 1189 Bq m^−3^ (range from 65–3957 Bq m^−3^). Moreover, these surveys revealed that the significance of thoron had been underestimated in the past [15]. Thus, thoron should be more rigidly defined from the viewpoint of health risk. 

In India, RADUETs were used to measure radon and thoron concentrations in 62 dwellings (cement, brick and mud houses) of a high natural background radiation area on the southeastern coast of Odisha and 259 houses (cement and brick, and some wood houses) on the southwest coast of Kerala [34,35]. Detectors were hung 0.2–2.0 m from the wall and 0.3–1.6 m from the ceiling in the bedroom or living room and some in the dining room or storeroom. At Odisha, respective radon and thoron concentrations ranged from 2–333 Bq m^−3^ with a mean value of 91 Bq m^−3^ and below the detection limit to 1004 Bq m^−3^ with a mean value of 105 Bq m^−3^. In addition, the respective radon and thoron concentrations at Kerala (two periods of six-month measurements) ranged from 1–21 Bq m^−3^ with a mean of 6 ± 4 Bq m^−3^ and a range of 3–212 Bq m^−3^ with a mean of 31 ± 13 Bq m^−3^, respectively. These studies showed higher concentrations of thoron in comparison with radon in certain cases. 

In Cameroon, a total of 450 RADUETs were used to measure indoor radon and thoron concentrations in houses around mining and ore-bearing regions of Poli, Lolodorf, Betare-Oya and Douala [24,36,37,38,39]. The monitors were hung from the ceiling in the bedroom for two months at a height of 1.5–2.0 m from the floor and 0.5 m from the wall. The respective indoor radon and thoron concentrations ranged between 46–143 and 24–238 Bq m^−3^ in Poli, 27–937 and 6–700 Bq m^−3^ in Lolodorf, 88–282 and 4–383 Bq m^−3^ in Betare-Oya, and 31–436 and 4–246 Bq m^−3^ in Douala. The mean values were 82 and 94 Bq m^−3^, 97 and 160 Bq m^−3^, 133 and 92 Bq m^−3^ and 139 and 80 Bq m^−3^, respectively. The results showed higher concentrations of thoron in comparison with radon in some cases.

In Thailand, indoor radon measurements in dwellings of Chiang Mai were conducted using 110 RADUETs (55 houses) during one year where a high number of new cases of lung cancer had been found [40]. All monitors were placed in the selected houses at a height of 1.0–2.0 m and 0.2 m from the wall in the bedroom for all seasons (two periods of six-month measurements in 2015–2016). The results showed that the mean radon levels varied from 35–209 Bq m^−3^, with an overall mean of 57 ± 2 Bq m^−3^. Moreover, there was no relationship between the indoor radon levels and seasonal variations caused by the influence of climate change as the mean radon activity concentrations showed no significant difference (*p* = 0.76) between the two periods of measurement. On the other hand, the results showed there was a lower level of thoron than radon in all the dwellings.

In Japan the third nationwide indoor radon survey was conducted in 2007–2010 [41] at 3461 dwellings (wooden, concrete, other and unknown types), assigned by the Neyman allocation method, using RADUETs. Moreover, the seasonal adjustment for each house type was applied to calculate indoor radon concentrations. The mean indoor radon concentration was 14.3 ± 14.7 Bq m^−3^. The indoor thoron concentrations were also obtained in this radon survey. However, it is difficult to obtain a representative indoor/outdoor thoron concentration because the monitors used for the survey were installed at different distances from the wall surface [15,42,43]. Therefore, it should be noted that the main purpose of a radon–thoron discriminative monitor like the RADUET is to reduce the influence of thoron on the radon measurements.

In Kenya, the concentration levels of radon and thoron were measured using 46 RADUETs in mud-walled, metallic- or iron sheet-walled and stone-walled non-traditional houses in the Kilimambogo region [44] and 20 RADUETs in traditional earthen dwellings in the Mrima Hill region [45,46]. The monitors were set in the dwellings for a three-month period at a distance of ~0.2 m far from the wall and 2.0 m above the floor. The mean radon concentration levels in mud-walled, metallic-walled and stone-walled non-traditional houses were 67 ± 11 Bq m^−3^ (ranged: 37–126 Bq m^−3^), 60 ± 10 Bq m^−3^ (ranged: 42–163 Bq m^−3^) and 75 ± 10 Bq m^−3^ (ranged: 38–84 Bq m^−3^) respectively, while the mean thoron concentration levels in the corresponding non-traditional houses were 195 ± 36 Bq m^−3^ (range: below the detection limit-973 Bq m^−3^), 71 ± 24 Bq m^−3^ (ranged: below the detection limit-1130 Bq m^−3^) and 161 ± 31 Bq m^−3^ (ranged: below the detection limit—385 Bq m^−3^). Moreover, the mean radon concentration in traditional houses was 35 ± 17 Bq m^−3^ (range: 16–56 Bq m^−3^), whereas the mean thoron concentration was 652 ± 397 Bq m^−3^ (range: 132–1295 Bq m^−3^). This implied that if a non-discriminative detector was used, and if doors and windows were closed at night as happens in habited dwellings, the radon levels in the huts would be much higher than reported.

In Australia, long term measurements (70–90 days in spring and winter for seasonal variation) were conducted for radon and thoron using RADUETs in an historical metalliferous underground mine in North Queensland [47]. The discriminative monitors were used as the primary monitors in the 2015 program and as comparison and quality monitors between other passive radon monitors and active monitors in a 2016 program. All monitors were places between 0.05 and 2.5 m from the mine wall surface. The radon and thoron concentrations ranged between 60 and 390 Bq m^−3^ (mean: 140 ± 55 Bq m^−3^) and 140 and 2600 Bq m^−3^ (mean: 1070 ± 510 Bq m^−3^), respectively. The radon results showed negligible significant variation between sampling periods and the thoron results at the same sampling locations showed the same trends for both seasons. However, the thoron data in places of high thoron concentration fluctuated according to the monitor placement at different distances from the exhalation surface (wall). Thus, the optimal sampling distance for thoron should be below 0.5 m from the wall, as thoron concentration varies significantly at distances over 0.5 m from it.

In Canada, RADUETs were used in a preliminary survey of simultaneous radon and thoron measurements at 93 dwellings in Ottawa over a period of three months [48]. In this preliminary survey, the monitors were placed on the lowest floor (basement) of the house and at the distance of ~0.5 m from the structure wall and the basement floor to increase thoron detection ability. Moreover, a survey of residential radon and thoron concentrations was conducted for three months in 2012–2013 fall/winter (October to March) periods at 3215 homes in 33 metropolitan areas, which covered ~70% of the Canadian population [49]. Monitors were installed in the lowest lived-in level of the home where the member spends at least 4 h a day. Moreover, monitors were located on the wall at a height of 0.8–2.0 m from the floor and ~0.5 m from the ceiling and ~0.2 m from other objects so as to give normal airflow around them. Radon concentrations in this survey ranged from 7–2117 Bq m^−3^ with a mean of 104 Bq m^−3^, whereas the thoron concentrations ranged from <4–210 Bq m^−3^ with a mean of 8 Bq m^−3^. Furthermore, there was no correlation between indoor radon and thoron concentrations and thoron concentrations could not be predicted from widely available radon information.

In South Korea, the nationwide survey for radon and thoron was done using RADUETs in 2008–2009 at 1110 public buildings (schools and local governmental offices) [50]. The annual means of indoor radon and thoron concentrations were 61 ± 2 and 11 ± 3 Bq m^−3^, respectively. Moreover, RADUETs were also used for quality control of the results in a complete survey on indoor radon concentration and the seasonal variation of indoor radon at all subway stations in Daejeon, in the central part of Korea in 2007–2008 [51]. The measurements were conducted quarterly for one year at three points: the ticket offices, ticket gates and platforms of each station. The monitors were placed more than 0.3 m from ceilings or walls and were not placed in the vicinity of air-conditioners, ventilation ducts and electrical devices to avoid effects that might be causes of heating or cooling, static electricity and thoron interference. The mean radon concentration of all the stations was 34 ± 15 Bq m^−3^ (range from 9–98 Bq m^−3^).

In Slovenia, RADUETs were used to study indoor radon and thoron concentrations in different environments [52]. The radon/thoron discriminative monitors were installed for two months at 2 dwellings, 7 kindergartens, 35 elementary schools, 4 hospitals, 9 spas and a karst cave; monitors were about 1.0 m away from a wall and 1.0–1.5 m above the floor. Radon and thoron ranges were 10–6870 and <4–1361 Bq m^−3^, respectively. The average ratio between indoor thoron concentrations and radon concentrations was 0.40. A radon and thoron survey in Slovenia indicated that thoron concentration in the environments was not negligible in comparison to radon concentration.

In Hungary, RADUETS were applied to investigate radon and thoron concentrations at 35 one-story dwellings near a closed uranium mine for 5- and 10-month long-term measurements [53], and 75 dwellings and 7 workplaces in 5 Hungarian counties for 3-month exposition periods [54]. In order to measure thoron, the RADUETs were placed mostly in inhabited areas of the houses, such as bedrooms and living rooms, at 1.0–1.5 m distance from the walls in the former study and at 0.15–0.20 m distance from the walls in the latter study. The radon concentration of houses near the closed uranium mine was calculated by applying seasonal correction. The results showed that the mean radon concentration was 579 Bq m^−3^ (range: 37–2195 Bq m^−3^); in some cases, the thoron concentrations were not negligible and indicated that radon measurements which are sensitive to thoron could be misleading. However, the distribution of the mean radon concentration in five Hungarian counties was 79 Bq m^−3^ in 80 measurement places and below 100 Bq m^−3^ in 58 cases; only one case exceeded 200 Bq m^−3^. The mean thoron concentration was 31 Bq m^−3^ (range: 1–285 Bq m^−3^). For about 10% of the measured rooms, the thoron concentration exceeded 100 Bq m^−3^. Consequently, it could be considered that the influence of thoron on the radiation dose might be considerable and therefore thoron measurements would be necessary.

In the Republic of Srpska, the thoron concentration was measured using RADUETs for 12 months in 25 primary schools of Banja Luka [55]. The monitors were deployed in one room of each school at a distance of ~0.1 m from a wall, a height of 1.5–2.0 m above the floor and at least 0.5 m from the corners. The thoron concentrations were lower than 200 Bq m^−3^, mainly below 100 Bq m^−3^ (range: 7–198 Bq m^−3^) with a mean of 63 ± 8 Bq m^−3^. The mean ratio between indoor thoron concentrations and radon concentrations was 0.68.

In the Former Yugoslav Republic of Macedonia, the first national survey of indoor radon and thoron concentrations was performed with RADUETs in 300 dwellings during four consecutive 3-month periods for one year [56,57,58]. Devices were deployed in the living rooms or the bedrooms at a height of 1 to 1.5 m above the floor and ≥0.2 m from other objects and > 0.5 m from walls to ensure they were less sensitive to the distance effect from the walls. The mean values of indoor radon concentrations in winter, spring, summer and autumn were obtained to be 115, 46, 87 and 92 Bq m^−3^, respectively. The maximum concentration of radon and an annual mean were 1276 Bq m^−3^ and 84 ± 2 Bq m^−3^, respectively. The means of indoor thoron concentrations in winter, spring, summer and autumn were obtained as 90, 56, 30 and 52 Bq m^−3^, respectively. The seasonal corrected annual mean concentrations ranged from 3–272 Bq m^−3^, with a mean of 28 ± 2 Bq m^−3^.

In Serbia, the specific activities of radon and thoron were carried out using RADUETs at 63 dwellings in rural communities of Central Kosovo, North Kosovo and Prizren regions [59], and at 43 dwellings in Sokobanja municipality (southern Serbia) [60]. RADUETs were mainly placed in old houses without concrete floors that had been built using building materials from the local environment and they were placed in living rooms and bedrooms for three to five months by hanging on the walls to reduce the thoron measurement uncertainty. The mean indoor radon and thoron concentrations were 459 Bq m^−3^ (range: 14−1640 Bq m^−3^) and 128 Bq m^−3^ (range: 1–635 Bq m^−3^), respectively. In addition, RADUETs were deployed for measuring radon and thoron in soil gas [61] at 27 sites around Tent B (southwest of Belgrade). The radon concentration in soil ranged from 0.8–25 kBq m^−3^, whereas the thoron concentration ranged from 0.6–1.9 kBq m^−3^.

In Romania, the radon/thoron survey was made at 35 schools in the northwestern region using RADUETs for a 3-month measurement period (covering spring) in a room located on the ground floor [62]. All monitors were positioned ~0.3 m from the wall and 1.0 m above the floor, in consideration of the spatial distribution of thoron in the ground-floor room. The measured radon and thoron concentrations ranged from 31 to 414 Bq m^−3^ (mean: 215 ± 10 Bq m^−3^) and below the detection limit to 235 Bq m^−3^ (mean: 70 ± 3 Bq m^−3^), respectively.

In Ireland, RADUETs were used in 205 dwellings over a 3-month measurement period to monitor indoor thoron gas from 2007 to 2009 [63]. The monitors were suspended on a wall or another suitable surface in the living room and in the main bedroom. By fixing monitors on the wall, thoron concentrations drop rapidly with distance from its source in the walls or other room surfaces due to its short half-life. Thus, the maximum thoron concentration in a room could be obtained from this survey. The thoron concentration ranged from <1–174 Bq m^−3^ with a mean of 22 Bq m^−3^. The data are the actual measured concentrations and are not seasonally corrected. 

The applications of RADUET to radon and thoron measurements in regional and nationwide surveys are summarized in Table 1.

## 4. Discussion

Variations in reported values of indoor radon and thoron concentrations in the countries described in Section 3 can be clearly seen. These variations may be attributed to the construction materials and ventilation used, and the geological structures in different countries. However, it is important to note that for the nationwide and regional surveys on radon and thoron the sampled dwellings or places in each geographical area are selected in such a way as to be representative of the population of that geographical unit. 

In this section, monitored rooms, geometry of the measurement space within the room, and the measurement period are briefly discussed on the basis of knowledge derived from the surveys in Section 3 which could provide a clearer understanding and roadmap for radon isotope measurements. 

Monitored room: most of the RADUETs were located within the ground floors of inhabited rooms, such as living rooms, dining rooms, bedrooms and classrooms, in order to obtain a population representative of the distribution of radon and thoron concentration levels in each country. 

Geometry of the measurement space within the room: radon is quite homogeneously distributed in a room [64]. Therefore, the position of the RADUETs for radon measurement can be chosen in many convenient ways, such as over a wardrobe or a bookcase, provided that it is exposed to indoor air. On the other hand, thoron concentration is not uniform in the room and decreases exponentially with the distance from the source [65]. The thoron results were quite high in surveys of China (max: 3954 Bq m^−3^), India (max: 1004 Bq m^−3^), Kenya (max: 1295 Bq m^−3^), Australia (max: 2600 Bq m^−3^), Hungary (max: 285 Bq m^−3^), Republic of Srpska (max: 198 Bq m^−3^), Serbia (max: 635 Bq m^−3^), Romania (max: 235 Bq m^−3^) and Ireland (max: 174 Bq m^−3^) due to placement of the RADUETs at distances less than 0.5 m from the wall. On the other hand, RADUETs were installed far from the wall (~0.5–1 m) in surveys made in Canada, Slovenia and Cameroon so as to give normal airflow around them. Thus, the effect of the actual site selected should be taken into account when obtaining a representative value of indoor thoron. Moreover, the point of measurement should not be placed close to heating or cooling sources as was done in the South Korea survey to avoid the heat and cool effects on monitors. 

Measurement period: the durations of radon and thoron measurements were for a single 2- and 3-month exposure period in Cameroon, Kenya, Australia, Canada, Slovenia, Romania and Ireland. Moreover, surveys in some countries used RADUETs placed for long-term measurements such as 5 and 10 months in Hungary, 6 months in China, 5 months in Serbia and 12 months in the Republic of Srpska. Additionally, shorter consecutive periods have also been adopted in Japan and the FYR of Macedonia (four consecutive 3-month periods) or in India and Thailand (two consecutive 6-month periods). However, it has to be pointed out that, for the time variations of radon and thoron concentrations and seasonal variations, the duration of measurements should be 12 months in total to obtain the annual mean. Besides, seasonal correction factors can be expressed as a function of the measurement period and the starting month [66]. In the case of radon surveys, the annual mean radon concentration estimated with a 3-month measurement and a seasonal correction factor has a higher uncertainty compared with 12-month measurement results, because seasonal variations can differ for different residences and the correction factors are based on mean seasonal variations [67,68]. Seasonal variations can differ for different countries as well as for different regions within a country. Thus, the seasonal correction should be estimated for radon and thoron concentrations in each region or country to evaluate the annual mean radon and thoron concentrations.

In Table 1 it can be observed that there are higher mean thoron levels than mean radon levels in Asia (China and India), Africa (Cameroon and Kenya) and Australia, whereas there are higher mean radon levels than mean thoron levels in Canada and Europe (Slovenia, Hungary, Serbia and Romania). Therefore, special attention should be paid to using the radon–thoron discriminative monitor in places with high thoron levels to obtain reliable and accurate results. Moreover, if devices which are somewhat sensitive to thoron are used for a nationwide or regional radon survey, the placement of the device should not be too close to walls suspected to have a considerable exhalation of thoron.

## 5. Conclusions

In this paper the advantages of the radon–thoron discriminative monitor known as the RADUET were reviewed as well as the strategic plans for regional and national surveys in various countries. Most were regional surveys of indoor radon and thoron measurements; only Japan, Canada and South Korea carried out national surveys using RADUETs. Though most studies reported the data for indoor radon and thoron concentrations by RADUETs, the Japanese survey addressed only radon concentrations. The aim of using the RADUETs in the Japanese survey was to decrease the effect of thoron on the radon measurements. The indoor thoron measurement in many countries was as thoron exhalation from walls and other inside room surfaces due to the monitors being placed at a distance below 0.5 m from walls and room surfaces. Surveys of several countries measured mean radon and thoron concentrations in dwellings by placing RADUETs at distances of less than 0.5 m to 2.0 m away from walls. 

In most instances and especially in high radiation areas, the results indicated that indoor radon concentrations would be much higher than reported if a non-discriminative detector was used. Moreover, the radiation doses produced by thoron cannot be neglected because sometimes it can be a more significant contributor than radon. 

Studies in most countries installed the monitors in the bedroom or main living space of the residences, workplaces and schools for a period of at least two to three months. The actual duration of the measurements depended on the activity of radon and thoron in each area.

In particular, radon concentration in houses or buildings can vary and change swiftly due to several factors including seasonal changes. In this review, some researchers applied a seasonal correction factor for radon concentrations and only one suggested use of the correction factor from each season to estimate annual dose due to radon and thoron [57]. Therefore, thoron seasonal corrections, which are currently unknown and cannot be assumed to be as analogous to indoor radon seasonal corrections, should be studied. 

This review detailed the importance of discriminative monitors for radon isotopes and their utilization in various countries. In addition, it can be used as a source of supporting data for regional and national surveys of radon and thoron measurement plans. It is very important to discriminate between radon and thoron signals for accurate radon measurement and for future risk assessment derived from internal exposure due to radon isotope inhalation.

## Figures and Tables

**Table 1:** Summary of the results obtained using RADUETs for radon and thoron surveys in various countries.

Country	Study Area	Measuring Conditions		Radon Concentrations (Bq m^-3^)			Thoron Concentrations (Bq m^-3^)			Reference
		Distance from Wall (m)	Distance from Floor (m)	Min	Max	Mean	Min	Max	Mean	
China	Yangxi and Yangdong	~0.3	~1.8	27	476	124 ± 78	65	3957	1247 ± 1189	[18,33]
India	Odisha	0.2–2.0	0.3–1.6	2	333	91	<LLD *	1004	105	[34]
	Kerala			1	21	6 ± 4	3	212	31 ± 13	[35]
Thailand	Chiang Mai	0.2	1.0–2.0	35	209	57 ± 2	-	-	-	[40]
Japan	-	-	-	-	-	14.3 ± 14.7	-	-	-	[41]
South Korea	Public building	-	-	-	1004	61 ± 2	-	-	11 ± 3	[50]
	Subway station	>0.3	-	9	98	34 ± 15	-	-	-	[51]
Cameroon	Poli	0.5	1.5-2.0	46	143	82	24	238	94	[24,36,37,38,39]
	Lolodorf			27	937	97	6	700	160	
	Betare-Oya			88	282	133	4	383	92	
	Douala			31	436	139	4	246	80	
Kenya	Kilimambogo and Mrima Hill	~0.2	2.0	16	163	59 ± 6	<LLD *	1295	270 ± 100	[44,45,46]
Australia	North Queensland	0.05-2.5	-	60	390	140 ± 55	140	2600	1070 ± 510	[47]
Canada	Ottawa	~0.5 and 0.2 from other objects	~0.5–2.0	7	2117	104	<4	210	8	[48,49]
Slovenia	-	1.0	1.0–1.5	10	6870	-	4	1361	-	[52]
Hungary	Closed uranium mine	0–2.0	1.0–1.5	37	2195	579	1	285	31	[53,54]
Republic of Srpska	Banja Luka	~0.1 and 0.5 from corners	1.5–2.0	-	-	-	7	198	63 ± 8	[55]
FYR Macedonia	-	>0.5 and ≥0.2 from other objects	1.0–1.5	-	1276	84 ± 2	3	272	28 ± 2	[56,57,58]
Serbia	Central Kosovo, North Kosovo, Prizren and Southern Serbia	0	-	14	1640	459	1	635	128	[59,60]
Romania	The north-western part	~0.3	1.0	31	414	215 ± 10	<LLD *	235	70 ± 3	[62]
Ireland	-	0	-	-	-	-	<1	174	22	[63]

* LLD = Lower Limit of Detection.
